# Enrichment analysis of genetic association in genes and pathways by aggregating signals from both rare and common variants

**DOI:** 10.1186/1753-6561-5-S9-S52

**Published:** 2011-11-29

**Authors:** Wei Yang, C Charles Gu

**Affiliations:** 1 Division of Biostatistics, Washington University School of Medicine, Box 8067, 660 South Euclid Avenue, St. Louis, MO 63110, USA; 2 Department of Genetics, Washington University School of Medicine, Campus Box 8232, St. Louis, MO 63110, USA

## Abstract

New high-throughput sequencing technologies have brought forth opportunities for unbiased analysis of thousands of rare genomic variants in genome-wide association studies of complex diseases. Because it is hard to detect single rare variants with appreciable effect sizes at the population level, existing methods mostly aggregate effects of multiple markers by collapsing the rare variants in genes (or genomic regions). We hypothesize that a higher level of aggregation can further improve association signal strength. Using the Genetic Analysis Workshop 17 simulated data, we test a two-step strategy that first applies a collapsing method in a gene-level analysis and then aggregates the gene-level test results by performing an enrichment analysis in gene sets. We find that the gene set approach which combines signals across multiple genes outperforms testing individual genes separately and that the power of the gene set enrichment test is further improved by proper adjustment of statistics to account for gene-wise differences.

## Background

Although it is debatable whether individual common single-nucleotide polymorphism (SNPs) or multiple rare variants underlie common human diseases, the truth is probably somewhere in between in that both types of variants might be important. Opportunities to include rare SNPs in current genome-wide association studies (GWAS) are brought forth by new high-throughput sequencing technologies. However, lone rare variants with appreciable effect sizes at the population level are hard to find. Therefore existing methods to detect risk rare variants are mostly of the collapsing type; these methods aggregate effects from multiple markers. Popular collapsing methods include the combined multivariate and collapsing (CMC) method [1] and the weighted-sum test [2]. These methods combine the effects of many SNPs in a chromosome region (e.g., vicinity of a candidate gene) to enhance statistical power.

Another way to combine small effects is to use enrichment analysis to test exceptional enrichment of signals in predefined sets of variables (e.g., SNPs in genes or genes in biological pathways). In general, when multiple effects are combined, noise (SNPs or genes) may be included indiscriminately with true signals. To achieve good test power, it is essential to properly use enrichment analysis or collapsing so as to ensure the focus on true signals while mitigating the influence of noise.

Gene set enrichment analysis (GSEA) was first applied in gene expression analysis [3] and was later brought into GWAS analysis [4]. We previously developed an extension of GSEA, called variable set enrichment analysis (VSEA), for improved enrichment analysis in GWAS [5]. One of the main issues addressed by our VSEA extension is how to properly normalize the statistics for aggregation to compensate for their distributional differences that result from various gene or gene set sizes or complicated interactions. This issue becomes more important when both rare and common SNPs are included in the analysis. In this paper, we test a two-step strategy that first applies a collapsing method at the gene level and then aggregates the gene-level test results using an improved enrichment analysis. This two-step approach is applied to the Genetic Analysis Workshop 17 (GAW17) simulated data with knowledge of the underlying simulating model; we also test both the CMC and the weighted-sum tests for gene-level analysis and both VSEA and GSEA for gene-set-level enrichment analysis.

## Methods

### Step 1: collapsing methods for genes

For gene-level tests, we used two collapsing methods to aggregate signals from multiple markers in genes. The first method is the CMC method. In this approach, rare variants (minor allele frequency [MAF] ≤ 0.01) in a gene are collapsed into a single variant and tested in regression models together with other common variants [1]. We consider two variations of the CMC method when defining the new collapsed variant. CMC-1 defines a 0/1 indicator for the presence of any rare variants; CMC-count uses the count of copies of rare alleles.

The second method is the weighted-sum test [2]. In this approach all variants within the gene are collapsed into a single variant by weighting each SNP to adjust for allele frequencies. When this method is applied to quantitative traits, we also use two variations of tests to get the *p*-values. WeightSum1 is a permutation-based approach to test the significance of the cross product of the collapsed variant and trait; WeightSum2 regresses the trait against the collapsed variant.

For more details on the collapsing methods, readers may refer to the review paper by Dering et al. [6].

### Step 2: enrichment tests for gene sets

Gene set enrichment tests are used to evaluate the combined significance of sets of genes. The procedure was first proposed by Subramanian et al. [3] in the original GSEA method to study differential genome-wide expression profiles. The method tests whether a gene set is significantly enriched with differentially expressed genes by comparing the enrichment of association signals in a given gene set with that in gene sets drawn at random. For the present study, we perform the GSEA using the gene-level statistics derived from the collapsing tests as gene scores. For improved power, we apply our VSEA extension developed for GWAS; this analysis properly adjusts for the difference in gene score distributions for genes of various sizes and SNP frequency composition [5] by using information from permutation tests. We apply this test to assess the power of detecting the true gene set and the stability when noise is included or when some true signals are excluded.

Using the GAW17 data set, we performed all gene-based and gene set tests using Q2 as the phenotype of interest and using the unrelated-individuals sample (697 subjects). For the gene set analysis, 13 genes that contribute to the risk of Q2 make up a gene set. In addition, we formed three types of gene sets by adding 5, 10, or 15 randomly selected genes to the Q2 genes; we also formed two more types by excluding 5 and 10 genes from the Q2 genes; one reference gene set consisted of genes contributing to Q1 phenotype; and finally, 200 gene sets consisted of randomly selected irrelevant genes to assess the false-positive rate, all with comparable gene set sizes (ranging from 3 to 64 genes). Two thousand permutations were used for the enrichment tests. We completed analyses of the first 25 replicates of the simulated data for this report.

### False-positive rates and spurious genes

Other investigators have observed extensive false-positive rates (FPRs) using various statistical methods [7,8]. One of the major reasons for the high FPRs is long-distance linkage disequilibrium among SNPs. We follow the method of Luedtke et al. [8] to identify “spurious genes” resulting from long-distance linkage disequilibrium and examine analysis results after excluding these genes.

Of the 3,205 genes in the data set, 13 contain causal SNPs, leaving 3,192 nonrisk genes. We define a spuriously associated gene as any nonrisk gene that is identified as significantly (*p* < 0.05) associated with the phenotype in at least 16 of the 200 replicates for the gene-based collapsing methods. We found that 980 genes were spurious.

## Results

### Step 1: gene-based tests

The FPRs of the four gene-based tests are shown in Table 1. For all collapsing methods, the FPR is inflated (0.087–0.108). After excluding the spurious genes, the FPR drops, but it is still slightly inflated (0.052–0.60). The FPR is well maintained using permutations.

**Table 1:** False positive rates at a nominal significance level of 0.05 in step 1

	CMC-1	CMC-count	WeightSum1	WeightSum2
Irrelevant genes	0.087	0.087	0.108	0.086
Irrelevant genes excluding spurious ones	0.060	0.060	0.059	0.052
Permutations	0.050	0.050	0.035	0.050

False positive rates were calculated by counting significant values in either irrelevant genes or in 2,000 permutation tests.

The power to detect the 13 genes using the collapsing methods in step 1 is shown in Table 2. The overall power of using the two CMC tests is similar and is generally higher than or comparable to that of the weighted-sum methods. Two genes, *VNN1* and *VNN3*, are easily picked up at a significance level of 0.05, especially using the CMC methods. Four other genes that have fair power (≥0.4) are *GCKR*, *PDGFD*, *SIRT1*, and *SREBF1*.

**Table 2:** Power to detect the risk genes for Q2 in step 1

Gene	CMC-1	CMC-count	WeightSum1	WeightSum2
*BCHE*	0.36	0.28	0.04	<0.01
*GCKR*	0.52	0.52	0.64	0.52
*INSIG1*	0.08	0.08	<0.01	0.12
*LPL*	0.24	0.24	0.04	0.04
*PDGFD*	0.52	0.52	0.04	<0.01
*PLAT*	<0.01	<0.01	<0.01	<0.01
*RARB*	0.16	0.16	0.24	0.12
*SIRT1*	0.40	0.52	0.52	0.36
*SREBF1*	0.44	0.44	<0.01	<0.01
*VLDLR*	0.04	0.04	<0.01	0.12
*VNN1*	0.88	0.92	0.96	0.96
*VNN3*	0.80	0.80	0.24	0.12
*VWF*	0.04	0.04	0.28	0.20

Power is shown at significance levels of 0.05.

### Step 2: gene set tests

We calculated the FPRs for the enrichment tests by using the proportion of signals in the 200 random gene sets at a significance level of 0.05 and averaging over 25 replications (Table 3). Both methods tend to give inflated rates to some extent whether spurious genes are included or not, although excluding them did produce better results (reducing FPR by 12.4% on average).

**Table 3:** False positive rates of VSEA at a nominal significance level of 0.05 in step 2

		Gene-based test			
		
	Method	CMC-1	CMC-count	WeightSum1	WeightSum2
Spurious genes present	GSEA	0.057	0.060	0.097	0.089
	VSEA	<0.001	0.086	0.080	0.080
Spurious genes excluded	GSEA	0.047	0.047	0.056	0.070
	VSEA	0.061	0.060	0.070	0.070

**Figure 1:**
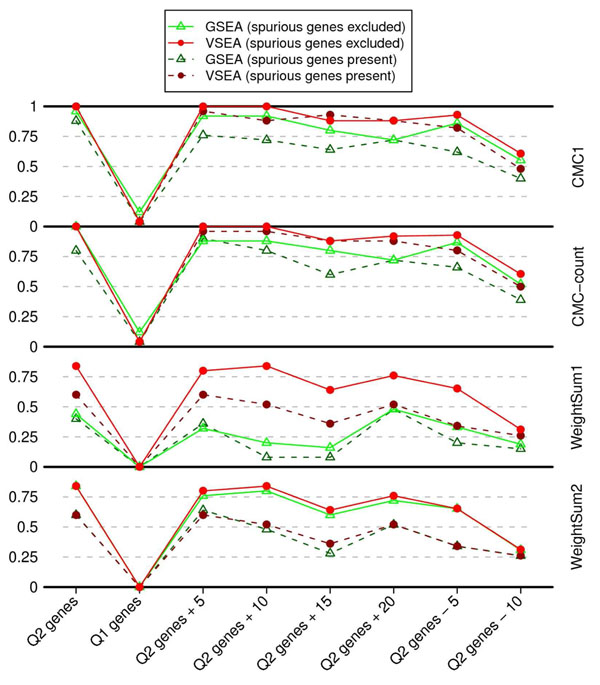
**Power of two types of gene set enrichment tests in step 2** Gene set enrichment analysis aggregates the results of gene-based tests for a group of genes. We tested the 13 genes contributing to the Q2 phenotype and used the genes for the Q1 phenotype as a negative reference. Noise was introduced to the Q2 genes by adding 5, 10, 15, and 20 genes. Also, in the last two gene sets part of the true signals was ignored by randomly excluding 5 or 10 risk genes. Power is shown for two types of enrichment tests: GSEA (without adjusting the gene-level test scores) and VSEA (gene scores adjusted). Tests were performed before (dashed lines) and after (solid lines) excluding spurious genes. These gene set tests were based on four gene-level tests (CMC-1, CMC-count, WeightSum1, and WeightSum2).

After adjusting the gene test statistics for their size and linkage disequilibrium structure in VSEA, we found that, when checking power, the gene set enrichment tests outperformed the straightforward GSEA in all situations (Figure 1). This result held no matter whether spurious genes were present or not, whether noise genes were included or true risk genes were left out, and whether the gene-level test used the CMC method or the weighted-sum method. When using the CMC methods and excluding spurious genes, VSEA achieves 100% power to detect the Q2 risk genes (*p* < 0.05), even when up to 10 random noise genes were included in the gene set. Moreover, the FPR of VSEA to find the reference Q1 risk gene set was always below the nominal 0.05.

When noise genes were included in the risk gene set or true risk genes were left out, both VSEA and GSEA suffered from power loss. For VSEA, when using the CMC methods and excluding spurious genes, the power remained as high as 0.88 when 20 noise genes were included, but power was reduced to about 50% when only 3 risk genes were included. Also, removing spurious genes improved the power of both VSEA and GSEA.

## Discussion and conclusions

We tested an improved method for enrichment analysis on top of gene-based collapsing methods for combining association signals across multiple genes. We compared the performance of the new method, which uses two collapsing methods to deal with the problem of small effects of rare variants, with two methods for gene set enrichment analysis and demonstrated the importance of normalizing gene-level test results. In the gene-level analysis, the CMC method performs slightly better than the weighted-sum method. It is worth noting that the weighted-sum method was originally proposed for case-control data with a rank sum test, and we tested association of the weighted sum with phenotype in order to extend it to quantitative trait analysis. At the gene set level, VSEA clearly outperformed direct application of conventional GSEA without normalization. The result shows that normalization of gene-based statistics is essential in gene-set-based enrichment analysis. Furthermore, the two-step approach of combining single rare variants across multiple genes clearly outperforms testing individual genes separately.

Data quality is an important issue no matter what method is used in the gene-level and gene-set-level tests. We observed inflated FPR in all our analysis methods when “dirty” data were used. However, after excluding spurious genes, the FPR dropped to close to a nominal level for both the gene-based tests and the gene set enrichment tests, and the FPR for the gene set methods was only slightly higher than the FPR for the gene-based tests. The fact that the FPR is still slightly inflated could be due to the complexity of the data. For example, we considered only the effect of spurious genes that have excessive correlation with true risk genes; the cryptic population structure observed by other GAW17 contributors was not addressed.

Finally, an important practical issue in successfully applying a gene-set-based strategy is to have a correct list of promising gene sets to begin with. Although many data-driven approaches have been proposed recently to create such a list, it is beyond the scope of this report and certainly warrants further investigation.

## Competing interests

The authors declare that there are no competing interests.

## Authors’ contributions

WY developed method, performed analysis, and drafted the manuscript. CCG developed the concept, participated in analysis, revised the manuscript critically, and gave final approval for publication. All authors read and approved the final manuscript.
